# Chirally Reversed Graphene Oxide Liquid Crystals

**DOI:** 10.1002/advs.202001269

**Published:** 2020-07-02

**Authors:** Yanjun Liu, Peiyi Wu

**Affiliations:** ^1^ State Key Laboratory of Macromolecular Engineering of Polymers Department of Macromolecular Science Fudan University Shanghai 200433 China; ^2^ State Key Laboratory for Modification of Chemical Fibers and Polymer Materials College of Chemistry, Chemical Engineering and Biotechnology & Center for Advanced Low‐dimension Materials Donghua University 2999 North Renmin Road Shanghai 201620 China

**Keywords:** chiral‐reversing, geometric confinement, graphene‐oxide liquid crystals, nanofluidics, polarization optics

## Abstract

Colloidal liquid crystals (LCs) formed by nanoparticles hold great promise for creating new structures and topologies. However, achieving highly ordered hierarchical architectures and stable topological configurations is extremely challenging, mainly due to the liquid‐like fluidity of colloidal LCs in nature. Herein, an innovative synchronous nanofluidic rectification (SNR) technique for generating ultralong graphene oxide (GO) liquid crystal (GOLC) fibers with hierarchical core‐skin architectures is presented, in which the GO sheet assemblies and hydrogel skin formation are synchronous. The SNR technique conceptually follows two design principles: horizontal polymer‐flow promotes the rapid planar alignment of GO sheets and drives the chiral‐reversing of cholesteric GOLCs, and in situ formed hydrogel skin affords some protection against environmental impact to maintain stable topological configurations. Importantly, the dried fibers retain the smooth surface and ordered internal structures, achieving high mechanical strength and flexibility. The linear and circular polarization potential of GOLC fibers are demonstrated for optical sensing and recognition. This work may open an avenue toward the scalable manufacture of uniform and robust, yet highly anisotropic, fiber‐shaped functional materials with complex internal architectures.

## Graphical abstract

Graphene oxide liquid crystal (GOLC) fibers with hierarchical core‐skin architectures are successfully fabricated using an innovative synchronous nanofluidic rectification technique, in which the hydrogel skin formation and graphene oxide sheet assemblies are synchronous. The GOLC fibers possess a reversed cholesteric order and exhibit controllable optical appearances that can be used for optical sensing and recognition.

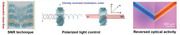

Colloidal liquid crystals (LCs) formed by nanoparticles are of great interest, since they offer unique structures and topologies for fundamental studies of soft matter physics and fabricating other new materials with complex hierarchical architectures.^[^ ^1^ ^]^ Of particular interest is the helical assembly of achiral flake nanoparticles, which can produce intriguing chiral LCs with both chirality and liquid crystalline behavior.

Graphene oxide (GO) is an oxygenated derivative of graphene, which can spontaneously form liquid crystalline suspensions with helical alignments.^[^ ^2^ ^]^ As a typical 2D colloid, graphene oxide liquid crystals (GOLCs) have gained increasing attention due to their remarkable electronic, optical, and mechanical properties.^[^ ^3^ ^]^ Nevertheless, many potential applications require that GOLCs possess long‐range ordered structures and long‐lived topological configurations. Such requirements are, however, extremely challenging in artificial colloidal LCs due to the liquid‐like fluidity of colloidal LCs in nature.

Many efforts have been deployed to stabilize the ordered alignment of nanosheets in artificial colloidal LCs using continuous external stimuli, such as electrical field,^[^ ^4^ ^]^ magnetic field,^[^ ^5^ ^]^ ultraviolet light,^[^ ^6^ ^]^ mechanical shearing,^[^ ^7^ ^]^ and chemical surface‐anchoring.^[^ ^8^ ^]^ These approaches only create GOLCs with a monotonic aligned organization. To date, there are no feasible and effective strategies for directly fabricating large‐scale GOLCs with long‐range helical systems and uniform and robust morphology. Gao et al. proposed a wet‐spinning strategy to manufacture macroscopic GOLC fibers with simultaneous lamellar ordering and helical frustrations,^[^ ^2^, ^9^ ^]^ however, the ordered internal structures were not retained in the dried fibers. The wrinkled surfaces and a large number of internal defects may have a severe impact on electrical and mechanical properties.^[^ ^10^ ^]^ Additionally, the GOLCs with controllable chirality have not been reported previously, because of no approach for easy, fine, and continuous control of the alignment direction of GO sheets in a fluid‐phase assembly process.

In this work, we propose an innovative synchronous nanofluidic rectification (SNR) technique to fabricate ultralong macroscopic GOLC fibers with hierarchical cholesteric liquid crystal (LC) architectures. Compared to extrusion,^[^ ^11^ ^]^ hot or cold‐drawing^[^ ^12^ ^]^ and wet‐spinning,^[^ ^2b^ ^]^ this new technique for fabricating fiber‐shaped materials with complex internal architectures is simple and scalable without the need for additional stimulus and tools. In particular, the SNR technique adds horizontal polymer‐flow within the ejected stream to promote the rapid planar alignment of GO sheets along the cylindrical radial direction and drives the rigid rotation of GO sheets to form chirally reversed cholesteric LCs, simultaneously. The hydrogel skin formed by the fast gelation of the polymer on the surface of the ejected stream can maintain the ordered hierarchical LC architectures with a long‐lived 3D topological configuration in the microtube space. More importantly, the dried fibers could retain the smooth surface and ordered internal structures, achieving high mechanical strength and flexibility. Besides, the GOLC fibers also exhibit controllable optical appearances that can be used for optical sensing and recognition.

**Figure** **1** illustrates the scalable fabrication of GOLC fibers with hierarchical core‐skin architectures using the SNR technique in which the hydrogel skin formation and GO sheet assemblies are synchronous. The SNR technique conceptually follows two design principles that are used in nature to produce complex hierarchical architectures through the integration of fundamental building blocks.^[^ ^13^ ^]^ First, the in situ formed hydrogel skin affords some protection against hydrodynamic shear impact. For conventional liquid–liquid interfaces, the ejected stream will be thinned into droplets owing to Plateau–Rayleigh instabilities,^[^ ^14^ ^]^ however, in our system, calcium alginate (Ca‐Alg) hydrogel skin could be formed rapidly on the surface of the ejected stream based on the fast cross‐linking reaction of sodium alginate (Na—Alg) and Ca^2+^ ions,^[^ ^15^ ^]^ arresting shape change and providing a stable and continuous assembly environment. Second, the SNR technique adds horizontal polymer‐flow within the ejected stream, together with the vertical central stream to form orthometric shear‐flow for the microfabrication of internal hierarchical LC architectures, which is in sharp contrast with the traditional drawing or falling stream that only has vertical shear‐flow.^[^ ^2^, ^16^ ^]^

**Figure 1:**
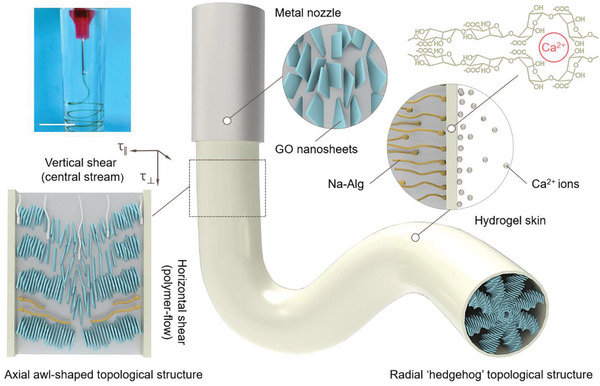
Schematic illustrations of flow‐induced hierarchical assembly of GOLC fibers. A digital photograph of the uninterrupted generation of GOLC fibers (upper‐left corner). Scale bars, 1 cm. Inset in the lower‐left corner, horizontal shear (*τ*_ǁ_): horizontal polymer‐flow‐driven alignment of GO sheets along the cylindrical surface and vertical shear (*τ*_⊥_): vertical central stream‐driven rotation of a cholesteric order along the GOLC fiber axis. Inset in the upper‐right corner, rapid formation of hydrogel skin on the surface of the ejected stream based on a fast cross‐linking reaction of Na—Alg and Ca^2+^ ions. The long‐lived 3D topological configurations within GOLC fibers consist of an axial awl‐shaped topological structure and a radial “hedgehog” topological structure.

As a typical experiment, 0.2 wt% GO suspensions containing 0.5% (*m/v*) Na—Alg and 5% (*m/v*) glucose were ejected into 50–100 × 10^−3^ M CaCl_2_ coagulation solutions through a metal nozzle (inner diameter, 270 µm). A simple peristaltic pump can be used to control the ejected speed at 0.5–2.0 mL min^−1^. Extrusion of the GO suspensions through the metal nozzle gives rise to shear and extensional flow fields that align the GO sheets in the direction of the ejected stream.^[^ ^11^ ^]^ As the GO suspensions exit the metal nozzle, traction forces exerted by the Ca^2+^ ions in the coagulation solution drive the outward movement of Na—Alg and then form a solid‐like hydrogel skin at the liquid–liquid interfaces. Meanwhile, polymer flow formed from the movement of Na—Alg induces the helical alignment of GO sheets from outward to inward along the cylindrical radial direction. As a result, the GOLC fibers possess hierarchical core‐skin architectures, in which a Ca‐Alg hydrogel skin encloses a colloidal cholesteric LC core.

The core‐skin structure was experimentally verified by the optical micrograph of GOLC fibers as shown in Figure S1,Supporting Information, where a bright‐field image of a 500 µm diameter GOLC fiber has a uniform and continuous hydrogel skin with a thickness of ca.15 µm. A representative scanning electron microscopy (SEM) image of the radial section of dried GOLC fibers is shown in Figure S2, Supporting Information. The distinct skin layer was also observed, consistent with the corresponding core‐skin structure observed by the optical microscope. As a result of the circular confinement of the hydrogel skin, GO cholesteric order in the colloidal core is radially organized into a radial “hedgehog” topological structure. Since Ca^2+^ ions jam rapidly at the interface of the ejected stream to increase interfacial resistance, leading to a velocity difference between the hydrogel surface of the GOLC fibers and their colloidal interior, the internal GO sheets can still move forwards when the external GO sheets are anchored on the hydrogel interface. Thus, the GO cholesteric order within the hydrogel skin skews along the axis of the ejected stream to form an axial awl‐shaped topological structure. The optical micrograph image shows distinct awl‐shaped LC textures along the axial direction, clearly indicating the axial structure of GOLC fibers.

In a stable environment, the GOLCs adopt a left‐handed helical alignment that has been reported in previous studies.^[^ ^2b^ ^]^ Here, by contrast, the chiral‐reversing of GOLCs leads to form right‐handed cholesteric LCs in dynamically microchannel confinement by polymer‐flow shearing, as illustrated in **Figure** **2**a. For cholesteric LCs, circular dichroism (CD) is an effective tool to accurately detect the selective reflection coefficients for right‐ and left‐handed circularly polarized light (Figure 2b).^[^ ^17^ ^]^ The chirality of cholesteric LCs in the GOLC fibers was confirmed by CD spectra analysis (Figure S3, Supporting Information), as seen in Figure 2c. It is important to mention first that the additive glucose does not affect the CD peak position and CD inversion of the structural chirality of GOLCs (Figure S4, Supporting Information). The GO solution exhibits a very weak positive CD peak that covers a wide wavelength range, implying a left‐handed helical alignment of GO sheets. On the contrary, both the GOLC fibers‐1(flow rate, 0.5 mL min^−1^) and GOLC fibers‐2(flow rate, 2 mL min^−1^) obtained at different flow rates exhibit the distinct negative CD peaks, a strong indication of the chiral‐reversing of cholesteric LCs in our GOLC fibers. Notably, a new strong negative CD peak appears in the CD spectrum of the GOLC fibers‐2 at the wavelength position of 320 nm, compared to the GOLC fibers‐1 with only one negative CD peak at the wavelength position of 549 nm. We attributed this new peak to the compact arrangement of cholesteric LCs in the marginal stream induced by the increased shear resistance of the ejected stream at a high flow rate. Previous studies have shown that CD peak position is gradually blue‐shifted with decreasing pitch.^[^ ^18^ ^]^ This trend was verified by comparing polarization optical microscopy (POM) images of the GOLC fibers‐1 and the GOLC fibers‐1 under crossed polarizers with a *λ* plate.

**Figure 2:**
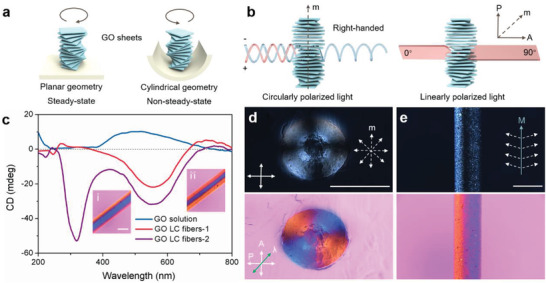
3D topological configurations within GOLC fibers. a) Schematic illustration showing a left‐handed GO cholesteric order in a stable planar geometry and a right‐handed GO cholesteric order in a dynamic cylindrical geometry. b) Schematic illustration showing the light path change of circularly and linearly polarized light through a right‐handed cholesteric GOLC order. c) CD spectra of GO solution (0.2 wt%), GOLC fibers‐1 and GOLC fibers‐2 obtained at the ejected speed of 0.5 mL min^−1^ and 2 mL min^−1^, respectively. Inset in c: (i) POM‐*λ* image of the GOLC fibers‐1 and (ii) POM‐*λ* image of the GOLC fibers‐2. Scale bars, 500 µm. d) POM image of the radial section of GOLC fibers with a Maltese cross pattern, and corresponding POM‐*λ* images with an alternating red and blue Maltese cross pattern. Inset in d: radial cholesteric axis field schematic. (arrow P, A, and *λ* represent the axis of the polarizer, analyzer, and *λ* plate, respectively). e) POM image showing a bright‐dark‐bright fringe along the axial direction of GOLC fibers, and corresponding POM‐*λ* images with an axial red‐blue fringe. Inset in e: an awl‐shaped cholesteric axis field schematic (m represents the direction of the axis of GO cholesteric order, M represents the axis of GOLC fibers). Scale bars, 500 µm.

The GOLC topological configurations were verified using POM and POM‐*λ*, as shown in Figure 2d and e. An essential feature of cholesteric LCs is that the periodical helical structures twist linearly polarized light when the axis (**m**) of cholesteric LCs has an inclination angle relative to the light plane (Figure 2b and Figure S5, Supporting Information). When observed by the POM with a *λ* plate, the right‐tilted cholesteric LCs exhibit vivid blue and the left‐tilted cholesteric LCs exhibit vivid red (Figure S6, Supporting Information), owing to the 530 nm optical path difference of the *λ* plate.^[^ ^19^ ^]^ Here we focus on the two representative POM images: radial and axial direction. The radial section of GOLC fibers exhibits the classic Maltese cross pattern corresponding to a radial “hedgehog” topological configuration with a central topological charge of +1 (Figure 2d). Note that the radial section has a distinct point defect with the radius ≈75 µm, which is in agreement with the size of GO sheets (Figure S7, Supporting Information). A black stripe parallel to the GOLC fiber axis (**M**) is visible in the POM image of the GOLC fibers, consistent with the corresponding point defect. Moreover, the POM‐*λ* image of the axial direction shows the red and blue stripes on both sides of the **M**, clearly indicating the axial awl‐shaped topological configuration. Therefore, we can conclude that a 3D topological architecture within GOLC fibers consists of chirally reversed cholesteric LC configurations, which possesses a radial “hedgehog” topological structure and an axial awl‐shaped topological structure. More importantly, the 3D topological architecture is very stable because of the protective effect of the robust hydrogel skin. As shown in Figure S8, Supporting Information, the distinct core‐skin structure of the GOLC fibers remains unchanged when they are immersed in a coagulation solution for 5 days.

The effect of GO concentration on the cholesteric LC configurations was investigated to further confirm the self‐assembly behavior of GO sheets in a dynamically microchannel confinement. **Figure** **3**a–c exhibits the representative Maltese cross patterns: sheath‐like cross pattern, hollow cross pattern, and solid cross pattern, a strong indication that the thickness of the GOLC layer increases with increasing GO concentration. This trend was clearly illustrated in the corresponding topological configuration schematic. Furthermore, the LC sheath is continuous along the internal surface of the GOLC fibers formed by 0.08 wt% GO solution (Figure S9, Supporting Information). This suggests that the self‐assembly direction of GO sheets is from outward to inward within the cylindrical geometry because of the concentration gradient formed by horizontal polymer‐flow shearing.

**Figure 3:**
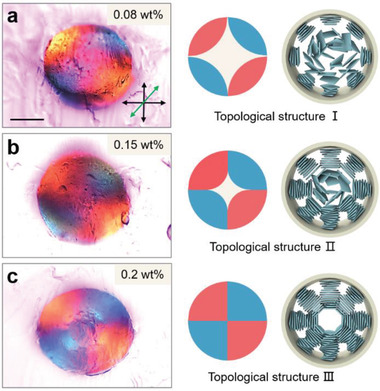
Radial LC configurations as a function of GO concentration. POM‐*λ* images, Maltese cross pattern schematic, and GOLC configuration schematic, from left to right. a) Sheath‐like Maltese cross pattern (0.08 wt%), b) hollow Maltese cross pattern (0.15 wt%), and c) solid Maltese cross pattern (0.2 wt%). Scale bars, 200 µm.

After determining the 3D topological configurations within GOLC fibers, we then turned our attention to the linear and circular polarization potential of GOLC fibers. As shown in **Figure** **4**a, because of the circular symmetry of the radial “hedgehog” topological configuration, the radial Maltese cross pattern remains unchanged when they are rotated 90° clockwise. By contrast, the axial direction exhibits a periodic color change from blue to red following the clockwise rotation (Figure 4b). At 180°, the position of red and blue stripes is reverse compared with the GOLC fibers at 0°. This result further verifies the axial awl‐shaped topological configuration, a strong indication that the GOLC fibers are unidirectional in a macroscale, which can be used as nanofluidic ionic cables for a directional ion transport.^[^ ^12a^ ^]^ Furthermore, the GOLC fibers can precisely twist linearly polarized light when the GOLC fibers have an inclination angle relative to the light plane (Figure 4c). The polar diagram of transmitted brightness is shown as a function of rotation angle in Figure 4d. The transmitted brightness of the GOLC fibers changes periodically with a bright‐dark transition per rotated 45°.

**Figure 4:**
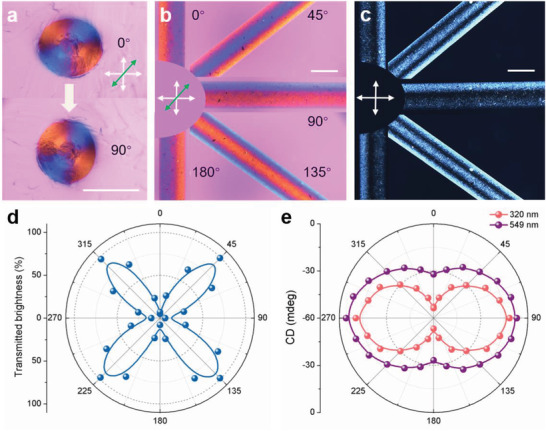
Responsive optical appearance and polarized light control. a) POM‐*λ* images showing the same Maltese cross pattern at 0°and 90°. b) POM‐*λ* images showing the optical appearance as a function of the rotation angle of GOLC fibers, and c) corresponding POM images. d) Transmitted brightness variation of linearly polarized light through the GOLC fibers as the angle changes. e) CD intensity variation of right‐handed circularly polarized light through the GOLC fibers as the angle changes at a wavelength position of 320 and 549 nm, respectively. Scale bars, 500 µm.

More importantly, compared to cholesteric cellulose films with changeless CD activity (planar geometry)^[^ ^20^ ^]^ and cholesteric LC microspheres without obvious CD activity (spherical geometry),^[^ ^21^ ^]^ the GOLC fibers exhibit a precisely tunable CD activity. The polar diagram of CD intensity is shown as a function of a rotation angle in Figure 4e. The CD intensity of the GOLC fibers changes periodically with a strong–weak transition per rotated 90°. The GOLC fibers with such a precise CD response can potentially act as optical control devices.

In addition to the tilted angle of cholesteric LC order under crossed polarizers, the pitch of cholesteric order is another parameter that can affect the optical appearance of GOLC fibers. As shown in **Figure** **5**a, the left‐tilted GOLC fibers showed vivid interference color changes from dull‐red to bright blue as the sample was stretched. Importantly, compared to as‐prepared GOLC fibers, the stretched GOLC fibers exhibit a reversed optical activity (Figure 5b). The main reason for this phenomenon is that the pitch decreases upon increasing of the elongation of the GOLC fibers, which leads to the change of a reflective visible‐light wavelength. CD spectra provided further confirmation of the reduction of the pitch in a stretching process. As shown in Figure 5c, the CD peak position is blue‐shifted from 550  to 350 nm with increasing the elongation, consistent with the corresponding color change observed by POM‐*λ*.

**Figure 5:**
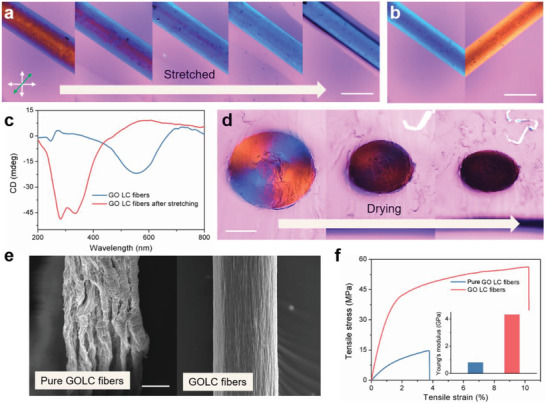
Controllable LC configurations and structural strengthening. a) POM‐*λ* images of GOLC fibers showing the color changes from dull‐red to blue upon stretching. b) POM‐*λ* images of the stretched GOLC fibers showing an opposite optical activity compared with as‐prepared GOLC fibers. c) CD intensity variation of the GOLC fibers before and after stretching. d) The Maltese cross pattern fades away as the shrinkage of GOLC fibers in the drying process. e) SEM image of dried GOLC fibers: pure GOLC fibers with wrinkled surfaces (without adding glucose) and GOLC fibers with smooth surfaces. f) Stress–strain curves of pure GOLC fibers and GOLC fibers under uniaxial tension, and corresponding Young's modulus (inset). Scale bars represent a,b) 500 µm, d) 200 µm, e) 50 µm.

The dependence of the LC configurations on water content is further demonstrated in Figure 5d. The Maltese cross pattern gradually disappeared upon the water evaporation and the GOLC fibers turned black in POM‐*λ* (Figure S10, Supporting Information), probably because CD activity disappears due to a parallel alignment of GO sheets along the fiber surface in the drying process.^[^ ^22^ ^]^ It should be noted that the shrinkage of a radial section is uniform because of the geometric confinement of the surrounding hydrogel matrix. Without an external constraint, the surface of GOLC fibers wrinkled sharply upon the loss of water due to the unbalance of the interfacial tension and anisotropic bulk elasticity (Figure 5e).^[^ ^23^ ^]^ The wrinkled surfaces and a large number of internal defects would limit their potential applications in energy storage, electronics, and nanocomposites. To minimize the surface distortion, we introduced 5% glucose as a thickener to increase the viscosity without affecting the LC configurations,^[^ ^24^ ^]^ thereby significantly reducing the anisotropic bulk elasticity and improving thermodynamic stability. As shown in Figure 5e, with the help of glucose, the GOLC fibers are shrunk uniformly during drying and the dried fibers exhibit a smooth surface. Furthermore, the mechanical strength and elongation of the GOLC fibers have a significant increase compared to the pure GOLC fibers. The mechanical strength and elongation of the GOLC fibers at break are at least four or two times higher than for the pure GOLC fibers, respectively. This suggests a simple route, structural strengthening, for fabricating high mechanical strength and tough fiber‐shaped materials by aligned elementary building blocks.

In summary, we present a strategy to produce ultralong GOLC fibers with hierarchical core‐skin architectures using polymer shear‐flow‐induced helical alignment of GO sheets within the hydrogel skin. Remarkably, the polymer shear‐flow drives the inverse rotation of GO sheets to form a chirally reversed GO cholesteric order. Based on the unique topological architecture, the CD activity of GOLC fibers can be precisely tuned with a rotated angle. Moreover, the GOLC fibers are unidirectional in a macroscale, which can potentially act as nanofluidic ionic cables for a directional ion transport. More importantly, the protective effect of a robust hydrogel skin enables the GOLC fibers with stable 3D topological LC configurations, which provides an attractive platform for fundamental studies of topological physics and as hard templates to fabricate other new materials with complex hierarchical architectures.

## Conflict of Interest

The authors declare no conflict of interest.

## Supporting information

Supporting Information
