# Supplementary material for: Chirally Reversed Graphene Oxide Liquid Crystals
Source: Adv Sci (Weinh). 2020 Jul 2;7(16):2001269. doi: 10.1002/advs.202001269 (PMC7435251; doi:10.1002/advs.202001269)
Supplement: Supplementary file 1 — Supporting Information [file ADVS-7-2001269-s001.pdf]

Copyright WILEY-VCH Verlag GmbH & Co. KGaA, 69469 Weinheim, Germany, 2020.

Supporting Information

Chirally Reversed Graphene Oxide Liquid Crystals

*Yanjun Liu and Peiyi Wu**

E-mail: peiyiwu@fudan.edu.cn

This file includes

Experimental Section

Characterizations

Figure S1. Optical micrograph of the GOLC fibers with a thin hydrogel skin

Figure S2. SEM image of the radial section of the dried GOLC fibers

Figure S3. Schematic illustration for CD detection of GOLC fibers

Figure S4. CD spectra of glucose and different GOLC fibers

Figure S5. Schematic illustration for POM-λ image acquisition of GOLC fibers

Figure S6. Relationship between the cholesteric order and the transmitted color

Figure S7. POM image of radial section of the GOLC fibers

Figure S8. The stability of GOLCs

Figure S9. POM images of GOLC fibers formed by 0.08 wt% GO solution

Figure S10. POM-λ images of GOLC fibers before and after drying

Experimental Section

Materials: Graphene oxide gel (GO, 1.6 wt%) with size of ~50 μm was obtained from Chengdu Organic Chemicals Co. Ltd., Chinese Academy of Sciences (http://www.timesnano.com). The pH of graphene oxide gel is 3-4. The number of graphene layers is 1-2. The percentage of oxygen atomic is 35.29%. Sodium alginate (Na-Alg, AR grade, 90%), D-(+)-glucose (AR grade), calcium chloride anhydrous (AR grade, 96.0%), The injection solutions were 0.08-0.2 wt% aqueous suspensions of GO containing 0.5% (m/v) sodium alginate and 5% (m/v) D-(+)-Glucose. The coagulation solutions was 50-100 × 10^{-3} M $CaCl_2$ solutions.

Preparation of graphene oxide liquid crystal (GOLC) fibers: 0.2 wt% GO suspensions were mixed with 0.5% (m/v) Na-Alg and 5% (m/v) glucose by magnetic stirring, then jetted into a 50-100 × 10^{-3} M $CaCl_2$ coagulation solutions by a sample microfluidic device at flow rates of 0.5-2.0 mL min^{-1}, and finally stored using glass culture dish containing 50-100 × 10^{-3} M $CaCl_2$ collection solution.

Characterizations

The self-assembly process of the GOLC fibers within the glass tube containing the collection solution was recorded by a regular digital camera. The optical images of the GOLC fibers were obtained by polarized optical microscopy under crossed polarizers with a λ plate (DM2500P, Leica). In the process of imaging, the GOLC fibers were immersed in a 50 × 10^{-3} M $CaCl_2$ solution to prevent the loss of water. The microstructures of the dried GOLC fibers were observed by field emission scanning electron microscope (Ultra 55, Zeiss). Optical activities of GO solution and the GOLC fibers were characterized by a circular dichroism spectrometer (A66, Applied Photophysics Ltd). In order to prevent the loss of water, the GOLC fibers were encapsulated into two pieces of quartz plate at different angles. The tensile testing of the dried GOLC fibers was performed on a universal electronic tensile machine (Instron 5966, Instron).

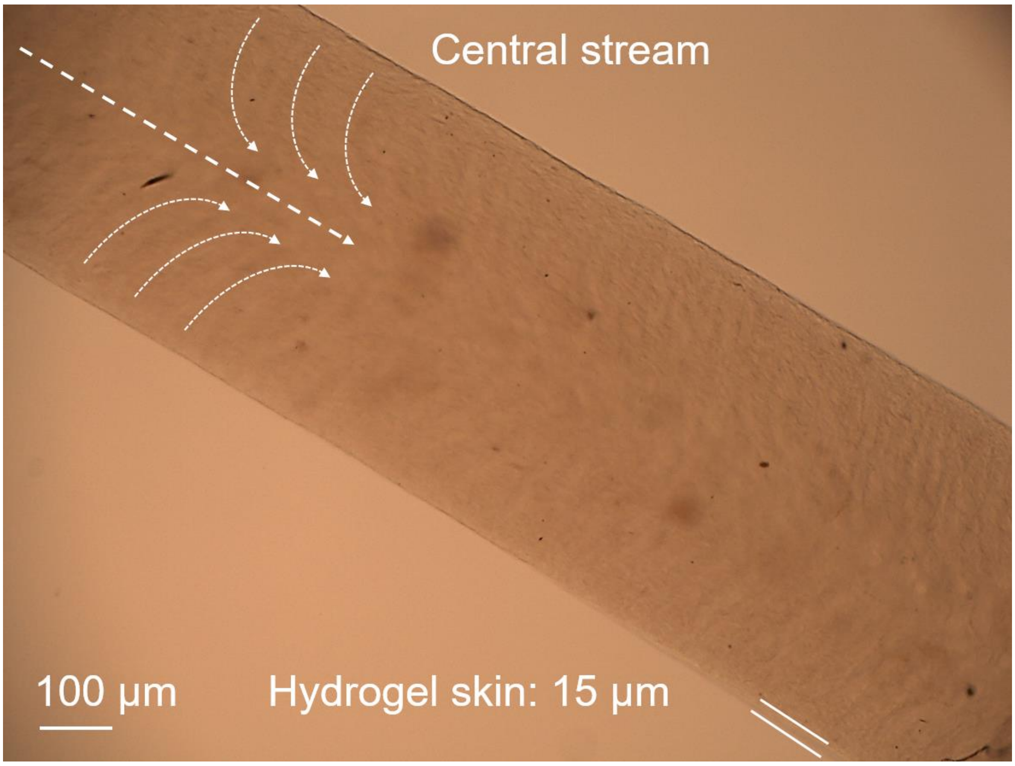

Figure S1. Optical micrograph of the GOLC fibers with a thin hydrogel skin. White arrows represent the shear path of central stream.

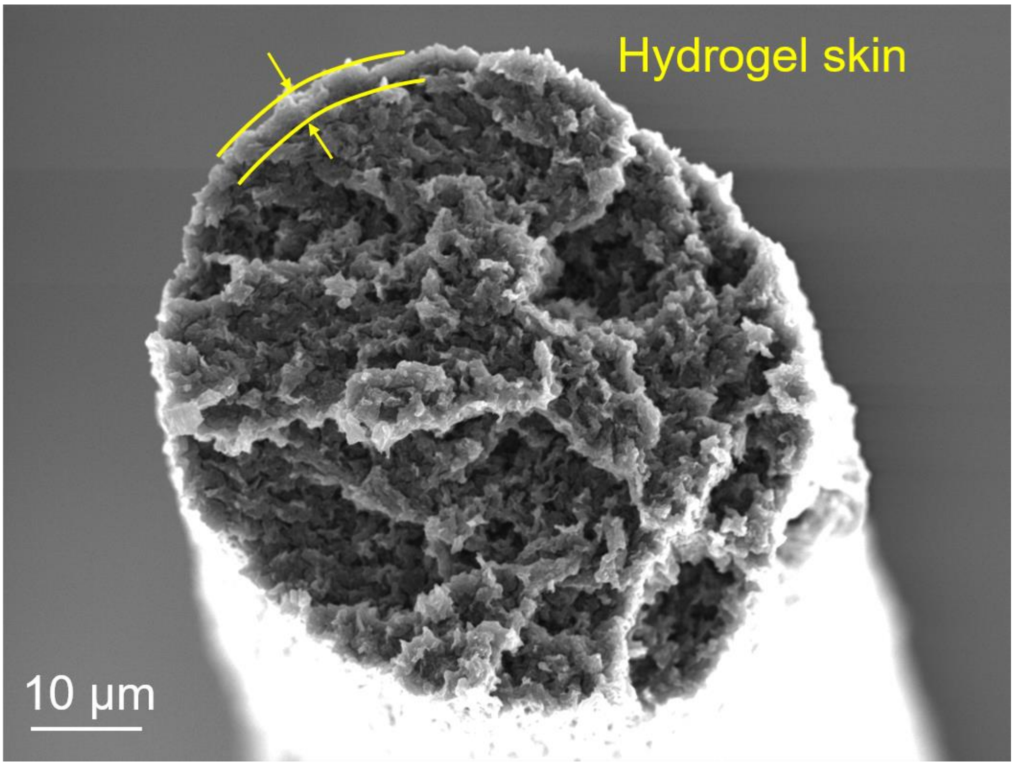

Figure S2. Scanning electron microscopy (SEM) image of the radial section of the dried GOLC fibers showing a distinct hydrogel skin.

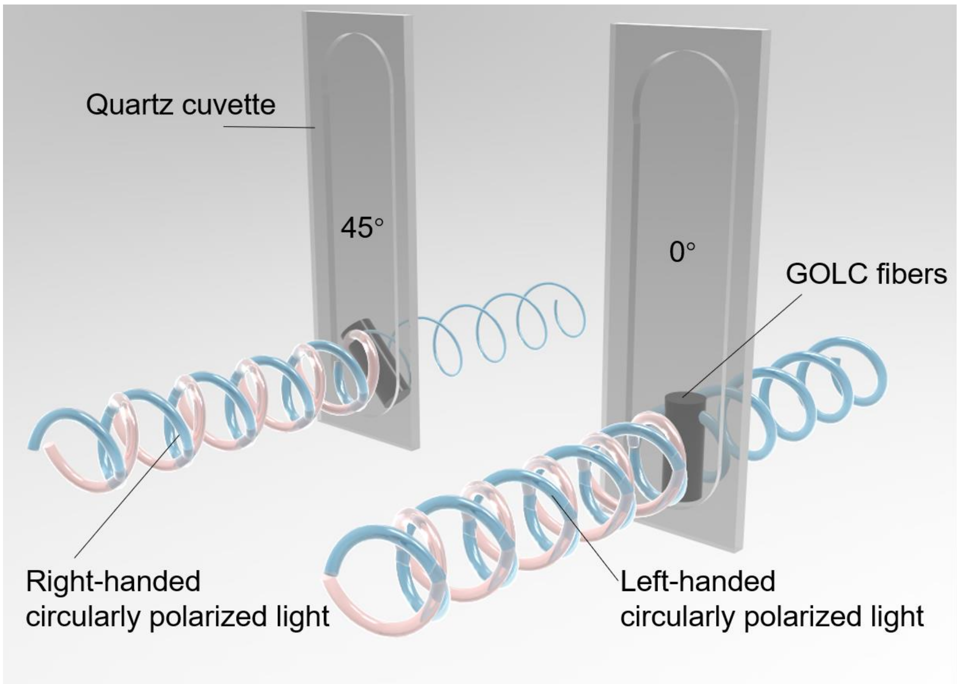

Figure S3. Schematic illustration for CD detection of GOLC fibers at different angle.

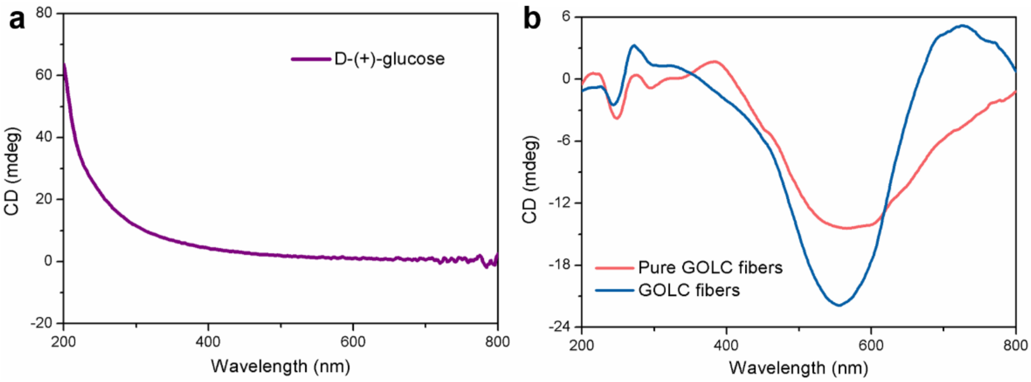

Figure S4. a) CD spectrum of 5% glucose. b) CD spectra of pure GOLC fibers and GOLC fibers containing 5% glucose.

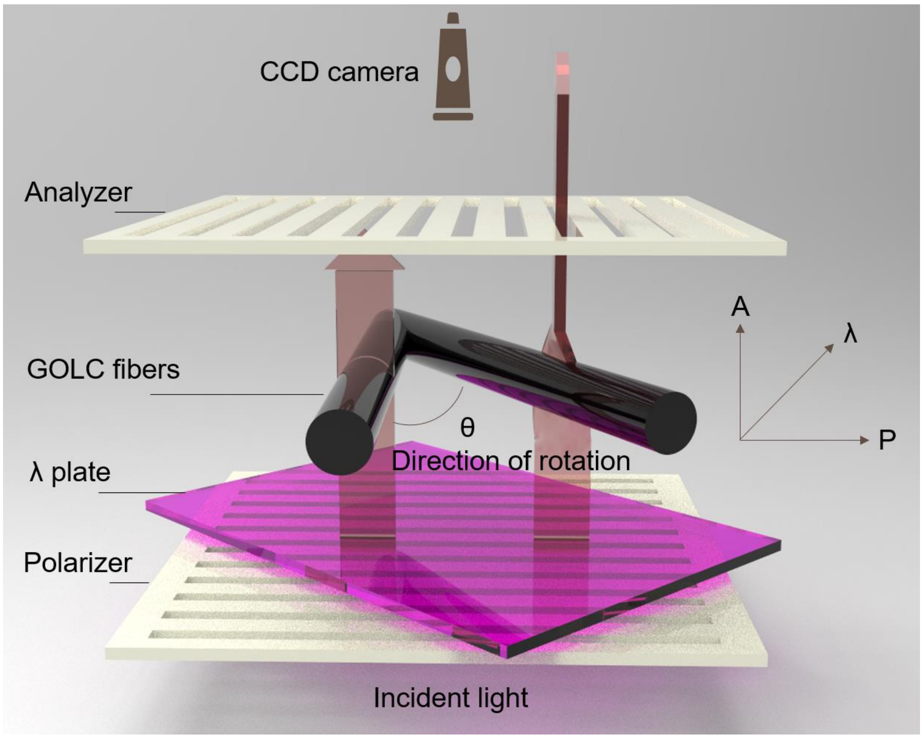

Figure S5. Schematic illustration for POM-λ image acquisition of GOLC fibers under crossed polarizers with a λ plate.

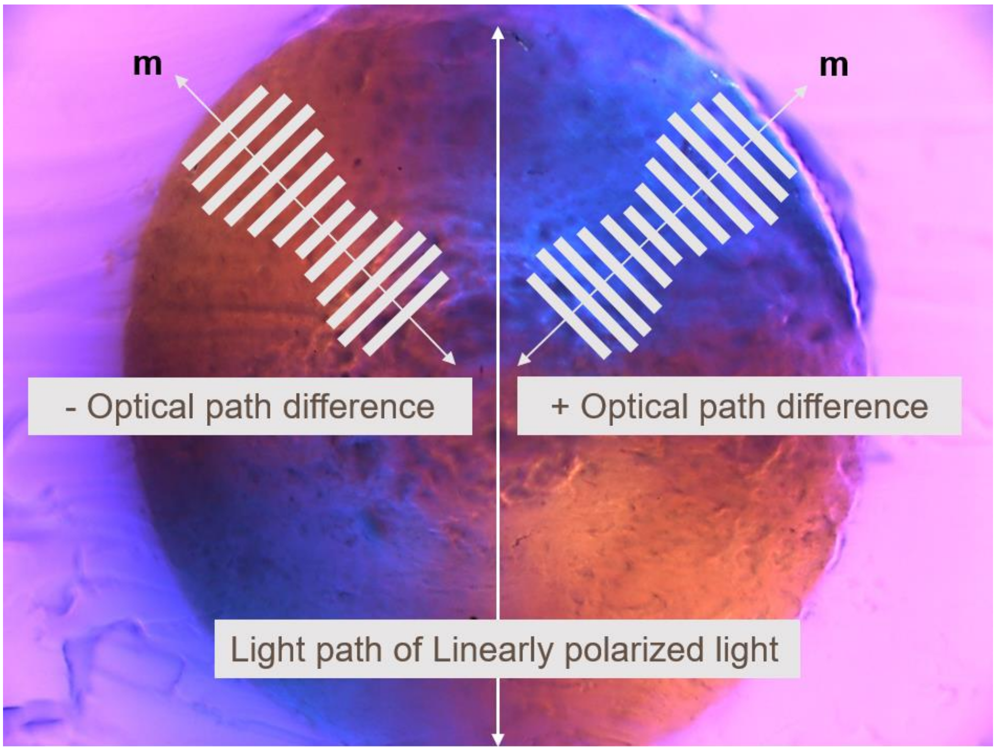

Figure S6. Relationship between the inclination angle of cholesteric order and the transmitted color of GOLC fibers under crossed polarizers with a λ plate.

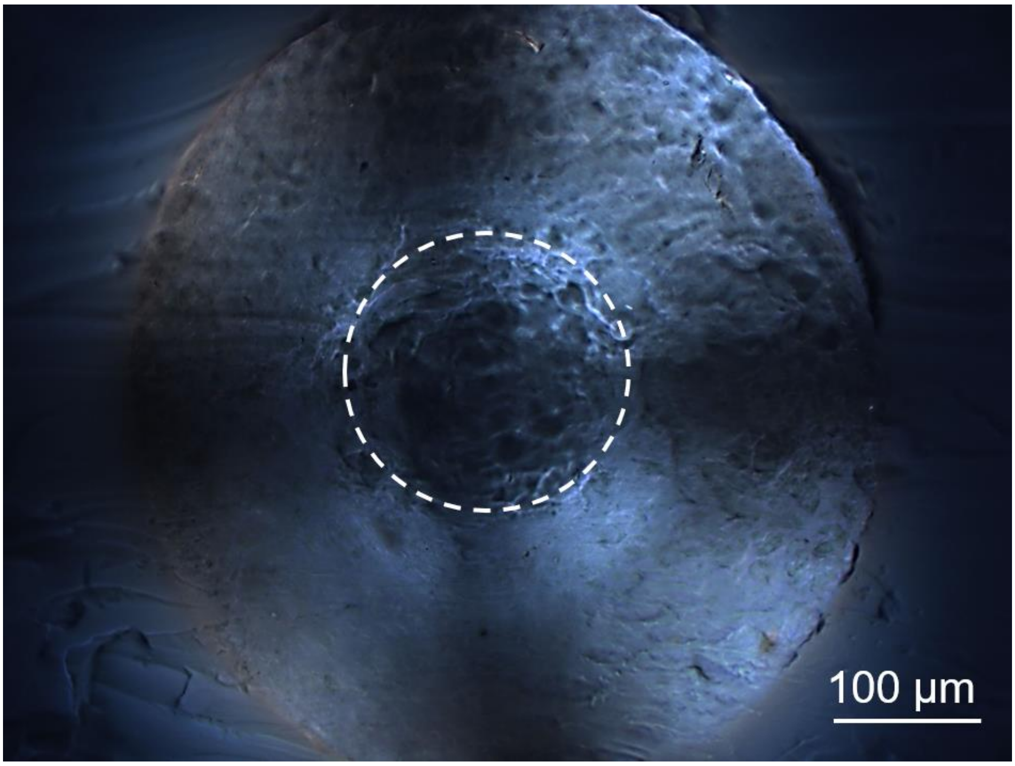

Figure S7. POM image of radial section of the GOLC fibers showing a distinct a radial-like singularity, known as a topological defect.

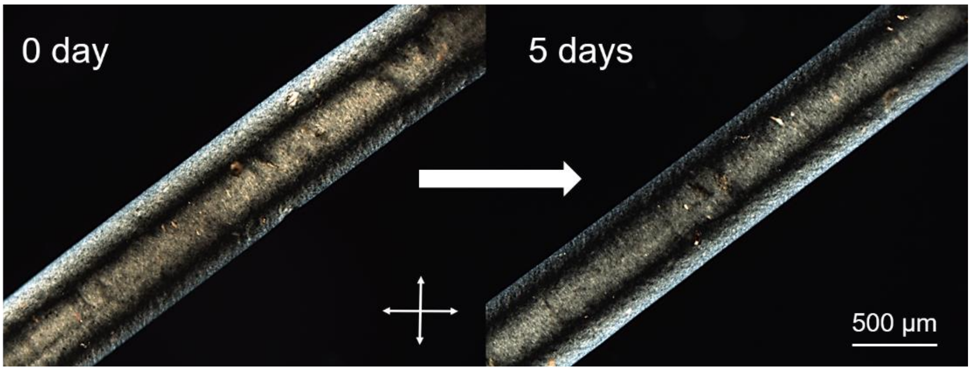

Figure S8. POM images of the GOLC fibers before and after being immersed in 50 × 10^{-3} M $CaCl_2$ coagulation solution for 5 days.

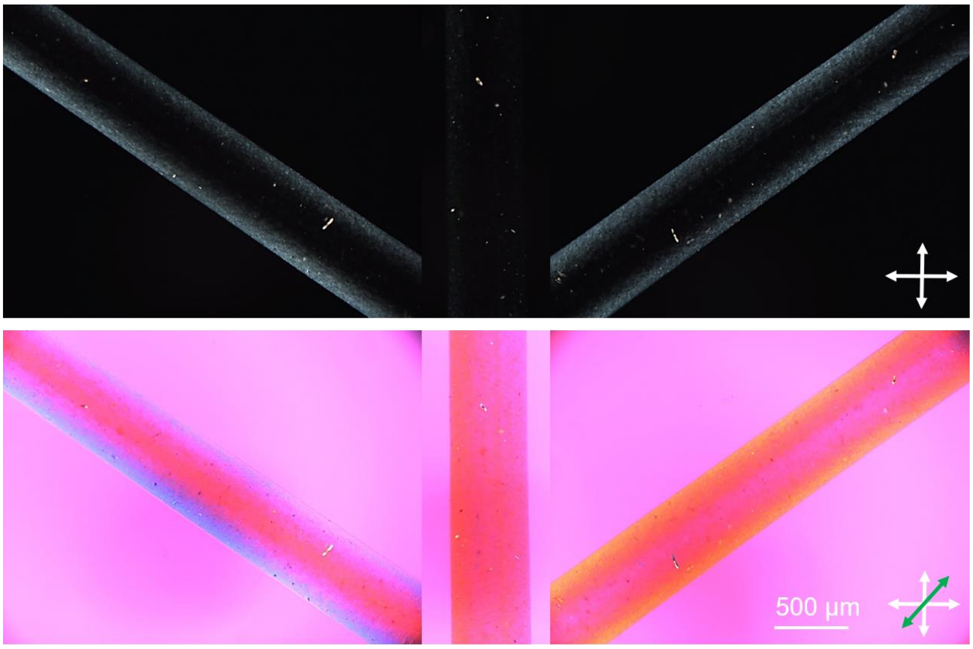

Figure S9. POM images of GOLC fibers formed by 0.08 wt% GO solution, and corresponding POM-λ images.

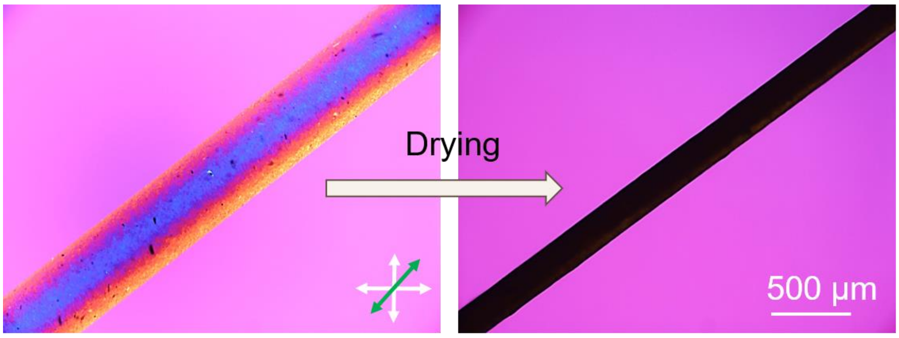

Figure S10. POM-λ images of GOLC fibers before and after drying.
